# GanedenBC^30™ ^cell wall and metabolites: anti-inflammatory and immune modulating effects *in vitro*

**DOI:** 10.1186/1471-2172-11-15

**Published:** 2010-03-24

**Authors:** Gitte S Jensen, Kathleen F Benson, Steve G Carter, John R Endres

**Affiliations:** 1 NIS Labs, 1437 Esplanade, Klamath Falls, OR 97601 USA; 2 AIBMR Life Sciences, 4117 S Meridian, Puyallup, WA 98373 USA

## Abstract

**Background:**

This study was performed to evaluate anti-inflammatory and immune modulating properties of the probiotic, spore-forming bacterial strain: *Bacillus coagulans*: GBI-30, (PTA-6086, GanedenBC30TM). In addition, cell wall and metabolite fractions were assayed separately to address whether biological effects were due to cell wall components only, or whether secreted compounds from live bacteria had additional biological properties. The spores were heat-activated, and bacterial cultures were grown. The culture supernatant was harvested as a source of metabolites (MTB), and the bacteria were used to isolate cell wall fragments (CW). Both of these fractions were compared in a series of *in vitro *assays.

**Results:**

Both MTB and CW inhibited spontaneous and oxidative stress-induced ROS formation in human PMN cells and increased the phagocytic activity of PMN cells in response to bacteria-like carboxylated fluorospheres. Both fractions supported random PMN and f-MLP-directed PMN cell migration, indicating a support of immune surveillance and antibacterial defense mechanisms. In contrast, low doses of both fractions inhibited PMN cell migration towards the inflammatory mediators IL-8 and LTB4. The anti-inflammatory activity was strongest for CW, where the PMN migration towards IL-8 was inhibited down to dilutions of 10^10^.

Both MTB and CW induced the expression of the CD69 activation marker on human CD3^- ^CD56^+ ^NK cells, and enhanced the expression of CD107a when exposed to K562 tumor cells *in vitro*.

The fractions directly modulated cytokine production, inducing production of the Th2 cytokines IL-4, IL-6, and IL-10, and inhibiting production of IL-2.

Both fractions further modulated mitogen-induced cytokine production in the following manner: Both fractions enhanced the PHA-induced production of IL-6 and reduced the PHA-induced production of TNF-alpha. Both fractions enhanced the PWM-induced production of TNF-alpha and IFN-gamma. In addition, MTB also enhanced both the PHA- and the PWM-induced expression of IL-10.

**Conclusion:**

The data suggest that consumption of GanedenBC30TM may introduce both cell wall components and metabolites that modulate inflammatory processes in the gut. Both the cell wall and the supernatant possess strong immune modulating properties *in vitro*. The anti-inflammatory effects, combined with direct induction of IL-10, are of interest with respect to possible treatment of inflammatory bowel diseases as well as in support of a healthy immune system.

## Background

An intact and properly functioning gastrointestinal (GI) system is of paramount importance in the maintenance of optimal human health. The GI tract plays important roles in nutrient absorption and assimilation, providing a protective barrier against invasion by harmful organisms, and training the immune system to distinguish between harmful and harmless substances. To maximize the absorption of nutrients obtained from the diet, the luminal side of the GI tract is organized into finger-like projections called villi. The surface area of the average GI tract has been calculated to be 300 m^2 ^and is lined by a single layer of epithelial cells. As a protective barrier against abrasion and to minimize contact of this epithelium by harmful organisms the GI tract is lined by a mucus layer consisting of polysaccharides. Beneath the epithelial cell layer are highly structured and active anatomical areas of our immune system, including the lamina propria and Peyer's patches. In order for the immune system to provide meaningful protection, it needs to be able to carefully discriminate between a large number and variety of antigens (proteins capable of inducing an immune response) that the GI tract is exposed to [1,2].

An optimal state of health can be adversely affected when any of these functions are compromised and can result in deficiencies due to malabsorption, infection and inflammation due to invasion by opportunistic organisms, or even autoimmune disease due to inappropriate immune system response to self. These perturbations can lead to a state of chronic low-grade inflammation. Chronic inflammation and gut microbiota imbalances are thought to be contributing factors to a variety of common diseases that have become serious public health problems including, but not limited to cardiovascular disease, obesity, cancer, diabetes, arthritis, depression, and inflammatory bowel diseases [3-7].

The GI tract is populated by as many as 10^14 ^microbes, which is many times greater than the entire number of cells comprising the human body. Since these organisms can be either commensal or pathogenic, the human body has had to develop effective strategies to maintain a balance such that the opportunistic pathogenic organisms are kept to a minimal number, thus limiting the inflammation and damage they can induce [2].

Probiotics are defined as viable microorganisms that can populate the GI tract in an active state and extend beneficial qualities to the host [8]. They play an important role in the health of the GI system by altering the environment, limiting the growth of pathogenic organisms, synthesizing nutrients, and increasing energy harvesting from the food we ingest. Species such as *Lactobacillus*, *Bifidobacteria*, and *Bacillus coagulans *are lactic acid producing bacteria, which can lower the pH, creating an environment that is not hospitable to many yeasts and bacterial species. Probiotic bacteria can also secrete antimicrobial compounds that are harmful to pathogenic organisms and thus limit their growth [9]. These bacteria also synthesize nutrients that are beneficial to the host and other beneficial gut organisms such as vitamin K2 and a variety of the B vitamins including folate and B12.

The immune system and the GI system are integrally connected [2]. It is at this interface between the lumen of the gut and the lamina propria that the immune system must make decisions regarding which substances are harmless and which are potentially deleterious. The foods we consume as well as opportunistic and beneficial microbes that occupy our gut are full of antigenic peptides and proteins capable of inducing immune responses. One function of the mucosal immune system is to mount an inflammatory immune response, when appropriate, to eliminate pathogens or to induce tolerance for those substances and organisms that are advantageous, such as food nutrients and probiotic bacteria [10]. Probiotic bacteria affect the adaptive and innate immune system by interacting with numerous cell types along the mucosa including B cells, T cells, regulatory T cells, monocytes, macrophages, NK cells and dendritic cells [9,11]. The immune response is determined by the environment, including cytokines, as well as the absence versus presence of inflammatory mediators.

Commonly consumed probiotic bacteria such as the *Lactobacillus *species are very sensitive to normal physiological conditions like the very low pH of the stomach, bile salts, and high temperatures. This creates challenges for the delivery of these bacteria to the intestines by oral consumption as well as challenges in manufacturing, shipping and storage conditions [12-14].

On the contrary, some strains of *Bacillus coagulans *are able to survive the extremes of heat, acidity of the stomach and bile acids. These characteristics make it an ideal probiotic due to the greater shelf-life stability and survivability to the intestines when consumed. *Bacillus coagulans *transiently occupies the gut for just a few days without repeated oral consumption. Spores of *Bacillus subtilis*, which is very closely related to Bacillus coagulans, have been shown to germinate and colonize the gastrointestinal tract in a murine model for a limited period [15]. The safety of long-term oral consumption of GanedenBC30TM (GBC30), was recently demonstrated [16].

The subject of this study is a proprietary strain of *Bacillus coagulans *(GBI-30, PTA-6086). In Asia, *Bacillus coagulans *is used in a number of commercially available products including a digestive biscuit for children and in natto (a fermented soy food) along with *Bacillus subtilus *[16,17]. GBC30, supplied by Ganeden Biotech, Inc. (Mayfield Heights, OH, USA) is a lactic acid producing bacteria that has been used as an ingredient in functional foods, dietary supplements, and medical foods. GBC30 is a gram-positive spore-forming rod that is aerobic to microaerophilic in nature and is manufactured as a pure cell mass consisting solely of a proprietary *B. coagulans strain*. Recent clinical studies have shown the efficacy of oral consumption of GBC30 for alleviating symptoms of irritable bowel syndrome [18] and inducing an increased immune response to viral challenge [19].

One aspect of probiotic research that has been questioned is whether the entire effect is due to cell wall components, or whether there are further advantages provided by live, metabolically active probiotic bacteria [20]. Much has been documented on the interaction of specific bacterial cell wall components with Toll-like receptors (TLRs) on immune cells', typically inducing innate immune defense mechanisms [21,22]. Gram negative bacteria predominantly engage the TLR-4 receptor through interaction with lipopolysaccharide present in the bacterial cell wall. Conversely, gram positive bacteria engage the TLR-2 receptor via interaction with lipoteichoic acids present in the bacterial cell wall [23]. However, the mechanism of action for atypical cell wall components, as well as the metabolites secreted by these organisms needs further elucidation [24]. Bacillus coagulans is not a typical inhabitant of the human gastrointestinal tract. Limited, but emerging research has been published on *Bacillus coagulans *as a probiotic and to the best of our knowledge this is the first study to investigate the differential effects of the isolated cell wall components compared with the bacterial metabolites. A broad panel of *in vitro *bioassays was perfomed to explore potential effects of GBC30 on different aspects of the immune system. This involved the study of both peripheral blood mononuclear cells (PBMC) and polymorphonuclear (PMN) cells.

## Methods

### Reagents

The following buffers and reagents were obtained from Sigma-Aldrich (St. Louis, MO): The T-cell mitogen Phytohemagglutinin (PHA), phosphate-buffered saline (PBS), RPMI-1640 culture medium, hydrogen peroxide 30% solution, Histopaque 1077 and 1119, fibronectin 0.1% from bovine plasma, and dimethyl sulfoxide (DMSO) 99.9%. The following reagents were obtained from Molecular Probes (Eugene, OR): 5-(and-6)-chloromethyl-2',7'-dichlorodihydrofluorescein diacetate, acetyl ester (CM-H_2_DCFDA), FluoSpheres^® ^carboxylate-modified microspheres 1.0 μm, and CyQuant^®^. The cytometric bead array for human Th1/Th2 cytokine kit II, CD69-FITC, CD25-FITC, CD107a-FITC, CD56-PE and CD3-PerCP were obtained from BD Biosciences (San Jose, CA). Sodium Azide (NaN_3_) was obtained from LabChem Inc. (Pittsburgh, PA). Low-binding 200 μm zirconium beads were obtained from OPS Diagnostics (Lebanon, NJ) and 0.2 μm cellulose acetate filters from Whatman (Florham Park, NJ). Multiscreen plates with 3.0 μm pore size for cell migration were obtained from Millipore (Bedford, MA). The *Bacillus coagulans *strain (GanedenBC^30™^) was obtained from Ganeden Biotech Inc. (Mayfield Height, OH).

### Preparation of Bacillus Coagulans Supernatant and Cell Wall Fractions

A sample of 2.0 g of GBC30 spores was placed into 25 mL PBS and heated at 70°C for 30 minutes. Spores were then centrifuged at 2400 rpm for 5 minutes, PBS removed and spores re-suspended in RPMI-1640 culture medium. The culture was incubated at 37°C for 2 days.

Preparation of GBC^30 ^culture supernatant (MTB) as a source of GBC30 metabolites: Following an initial spin at 3000 rpm for 15 minutes, the supernatant was removed and centrifuged further at 3500 rpm for 20 minutes. The supernatant was then filtered twice through a 0.2 μm cellulose acetate syringe filter and 250 μL aliquots stored at -20°C.

Preparation of GBC30 cell wall fragments (CW): The bacterial pellet from the initial centrifugation of the 2-day culture was processed through multiple bead milling and freeze/thaw cycles. In brief, the pellet was resuspended in 4 mL of PBS and 4 mL of 200 μm low-binding zirconium beads were added. One cycle of bead milling consisted of 60 one-second pulses of the bacteria/bead mixture on a vortex mixer. Five of these cycles were performed. The supernatant was removed from the beads and spun at high speed in 1.5 mL microcentrifuge tubes. The pellets were combined and re-suspended in 1 mL PBS and processed through 3 freeze/thaw cycles and placement in a sonication bath for an hour. The final solution was filtered through a 0.2 μm cellulose acetate filter and 250 μL aliquots stored at -20°C.

### Purification of peripheral blood mononuclear cells (PBMC) and polymorphonuclear (PMN) cells

Healthy human volunteers between the ages of 20 and 50 years served as blood donors upon informed consent, as approved by the Sky Lakes Medical Center Institutional Review Board (FWA 2603). Freshly drawn peripheral venous blood samples in sodium heparin were layered onto a double-gradient of Histopaque 1119 and 1077, and centrifuged for 25 minutes at 2400 rpm. The upper, PBMC-rich and lower PMN interfaces were harvested using sterile transfer pipettes into new vials, and washed twice with 10 mL PBS without calcium or magnesium by centrifugation at 2400 rpm for 10 minutes. The PBMC fraction contains the natural killer (NK) cell population and was used for testing of NK cell activity as well as lymphocyte proliferation and cytokine production.

### Phagocytosis assay

Evaluation of phagocytic activity was performed using human PMN cells. The choice of particles for phagocytosis was carboxylated FluoroSpheres (Molecular Probes, Eugene OR), due to their surface treatment that decreases nonspecific binding and facilitates uptake by phagocytes. These beads fluoresce in the yellow-green spectrum (505/515 nm). An aliquot of 0.05 mL FluoroSpheres was removed from the stock bottle into a 1.5 mL microcentrifuge tube and washed twice in PBS. FluoroSpheres were then re-suspended in 7.5 mL RPMI 1640. PMN cells were plated into 96-well plates in RPMI-1640 at a concentration of 2 × 10^6 ^cells/mL. Ten microliters of 10-fold serial dilutions of MTB or CW were added to test wells in quadruplicate, and PBS was added to control wells in quadruplicate. The plate was immediately centrifuged, and the supernatant removed. The cells were re-suspended in RPMI-1640 containing FluoroSpheres, and then incubated for 2 minutes with FluoroSpheres with continuous pipetting. The phagocytic activity was stopped by adding PBS with 0.02% sodium azide. Cells were washed twice in PBS with sodium azide to remove beads not ingested by the cells. Samples were transferred into vials for flow cytometry, ensuring the continued presence of sodium azide. Samples were acquired by flow cytometry immediately (FacsCalibur, Becton-Dickinson San Jose, CA). The analysis was performed using the FlowJo software (TreeStar Inc., Ashland OR). During analysis, electronic gating for the PMN population was performed using the forward and side scatter properties. The relative amount of phagocytosis within the PMN population in each sample was evaluated by the mean fluorescence intensity (MFI) for the green fluorescence. The MFI (green) for the untreated samples showed the relative amount of phagocytosis in the absence of MTB and CW. The MFI (green) for the MTB and CW treated samples were compared to untreated samples. Phagocytosis samples were assayed in triplicates and experiments repeated three times with PMN cells from three different donors.

### Reactive Oxygen Species (ROS) production by PMN cells

The production of ROS by PMN cells was tested as described previously [25]. Parallel samples of PMN cells were incubated at 37°C, 5% CO_2 _for 20 minutes, either untreated or with test products over a range of 10-fold serial dilutions (1:10, 1:100, 1:1000). The precursor dye DCF-DA, which becomes brightly green fluorescent upon exposure to free radicals, was prepared by adding 0.18 mL DMSO to a 0.05 mg aliquot of DCF-DA. A working solution of DCF-DA was then prepared by adding 0.01 mL stock to 10 mL PBS. The PMN cells were washed three times in PBS and then re-suspended in the DCF-DA working solution and incubated for 1 hour at 37°C. All samples, except for the untreated control samples, were then exposed to 167 mM H_2_O_2 _for a period of 45 minutes to induce ROS production. Samples were washed twice in PBS to remove the peroxide, and transferred to vials for flow cytometry. The DCF-DA fluorescence intensity in untreated versus H_2_O_2_-challenged cells was analyzed by flow cytometry. Data was collected in quadruplicate and experiments performed 3 times using cells derived from 3 different donors. The relative amount of ROS formation in PMN cells was evaluated by green fluorescence intensity.

### PMN cell random migration and chemotactic migration towards three chemo-attractants: f-MLP, IL-8 and Leukotriene B4

The PMN cell is a highly active and migratory cell type. The differential effect on PMN cell migration towards the bacterial peptide formyl-Met-Leu-Phe (f-MLP) and two different inflammatory chemo-attractants IL-8 and Leukotriene B4 (LTB4) were tested, as described previously [26]. The following experimental model was performed in quadruplicate in order to obtain data significance. Cells were incubated with 10-fold serial dilutions of GBC30 supernatant or cell wall fractions for 10 minutes in a polystyrene round-bottom tube before plating commenced. During this time the Millipore trans-well (3.0 μm pore size) migration plate was coated with 50 μg/mL Fibronectin for a period of 30 minutes. Chemoattractants and RPMI 1640 were then added to the appropriate bottom chamber wells of the trans-well migration plate in a volume of 150 μL: f-MLP (10 nM), Interleukin-8 (10 μg/mL), and Leukotriene B4 (10 nM). Fibronectin was removed from the top wells by aspiration before plating of cells. Fifty microliters of cells (1 × 10^6^/mL) were plated in the top chambers, and the top chamber plate was then lowered into the bottom plate and allowed to incubate overnight at 37°C. Quantification of the relative amount of migrated cells was performed by fluorescent CyQuant^® ^staining of the cells that had accumulated in the bottom chambers. Fluorescence intensity was quantified in a Tecan Spectrafluor fluorescence plate reader. Samples were assayed in triplicate or quadruplicate and experiments repeated at least 3 times with cells from different donors.

### Immunostaining for Natural Killer cell activation markers

Freshly isolated PBMC were distributed in a sterile U-bottom 96-well culture plates (NUNC, Denmark) and treated with serial dilutions of test products [27,28]. For activation of natural killer (NK) and natural killer T (NKT) cells the incubation time was 18 hours. Cells were transferred to V-bottom 96-well plates (NUNC Denmark) and washed in IF buffer (PBS containing 1% bovine serum albumin and 0.02% sodium azide). Cells were re-suspended in 0.05 mL IF buffer and monoclonal antibodies were added in previously established optimal quantities (CD3-PerCP, CD56-PE, CD69-FITC, and CD25-FITC: 8 μL/sample), and incubated in the dark at room temperature for 10 minutes. The cells were washed twice with an additional 0.15 mL of PBS with 0.02% azide. Following centrifugation and aspiration of the supernatant, the cells were re-suspended in 0.05 mL PBS with 0.02% azide and transferred to 5 mL polystyrene round-bottom tubes each containing 0.4 mL of 1% formalin. Samples were stored in the dark and acquired by flow cytometry within 24 hours using a FACSCalibur flow cytometer (Becton-Dickinson, San Jose CA). Analysis was performed using the FlowJo (Tree Star Inc., Ashland OR) software. Samples were assayed in duplicate and experiments repeated three times with cells derived from three different donors.

### Externalization of CD107a on NK cells in response to K562 tumor cells

The CD107a marker is constitutively expressed on the interior of lysosomes, and transiently expressed onteh cell surface of NK cells that are actively engaged in the killing of transformed cells [29]. Freshly purified human peripheral blood mononuclear cells (PBMC) re-suspended in RPMI 1640 were used for this assay. The cells were plated at 2 × 10^5^/well in round-bottomed 96-well micro-assay plates, and treated with serial dilutions of the test products in triplicate. Negative control wells in triplicate were left untreated. In addition, three wells containing PBMC alone and K562 cells alone served as negative controls for baseline CD107a expression. 1 × 10^6 ^K562 cells, an NK-cell sensitive tumor cell line widely used in NK cell cytotoxicity studies, were added to wells containing PBMC with product and untreated PBMC. The two cell types were loosely pelleted by a brief 30-second centrifugation at 2400 rpm followed by incubation at 37°C for 45 minutes. Cells were transferred to V-bottom microtiter plates for processing and staining. Cells were stained with CD3-PerCP, CD56-PE and CD107a-FITC. The expression of CD107a on the NK cells was determined by flow cytometry. The CD3 negative, CD56 positive NK cells were differentiated from the K562 cells based on forward and side scatter properties, and from other lymphocytes by electronic gating on CD3^-^, CD56^+ ^cells, followed by evaluation of fluorescence intensity for CD107a. Samples were assayed in triplicate and experiments repeated three times using cells derived from three different donors.

### Modulation of proliferation and cytokine production in response to PHA and PWM

Freshly purified PBMC re-suspended in RPMI 1640 supplemented with 10% fetal calf serum, L-glutamine (2 mM), penicillin (100 U/mL) and streptomycin (100 mg/mL) were plated in a U-bottom cell culture plate at a volume of 180 μL at a concentration of 1 × 10^6^/mL. Next, 20 μL of 10-fold serial dilutions of MTB and CW were added to the individual wells in triplicate. In a parallel set of wells, the combinatorial effect of MTB and CW with known mitogens was tested. Mitogens were added at a concentration of 5 μL of PWM (200 μg/mL) and 4 μL of PHA (2 μg/mL) to initiate proliferation. The plate was sealed with parafilm and was incubated at 37°C, 5% CO_2 _for 5 days. After 5 days the cells were transferred to a flat-bottom black 96-well plate and the relative cell numbers in each culture well quantified by CyQuant^® ^staining and a Tecan Spectrafluor fluorescence plate reader. Samples were assayed in triplicate and experiments repeated three times with cells derived from three different donors.

Supernatants from the 5-day lymphocyte proliferation cultures were harvested and relative levels of the 6 cytokines: IL-2, IL-4, IL-6, IL-10, TNF-α, and IFN-γ were measured using a flow cytometry-based bead array kit (CBA human Th1/Th2 cytokine kit II, BD Biosciences, San Jose, CA). Samples were assayed for the 1:100 dilution of MTB and CW. Samples were tested in duplicate according to the manufacturer's specifications, and data acquired immediately by flow cytometry, using a FacsCalibur flow cytometer (Becton-Dickinson San Jose, CA). The analysis was performed using the FlowJo software (TreeStar Inc., Ashland, OR).

### Statistical analysis

Statistical significance was tested using Student's t-test with *p *< 0.05 indicating a statistically significant and *p *< 0.01 a highly statistically significant difference between two data sets. Analyses were performed using Microsoft Excel. Only statistically significant *p *values are reported.

## Results

### Inhibition of Formation of Reactive Oxygen Species (ROS) by PMN cells

The PMN cell is involved in innate immune defense mechanisms, including the formation of ROS as part of both anti-bacterial and pro-inflammatory reactions. Treatment with complex natural products can cause signaling of pro- as well as anti-inflammatory mechanisms, leading to either enhancement or reduction of ROS formation.

**Figure 1:**
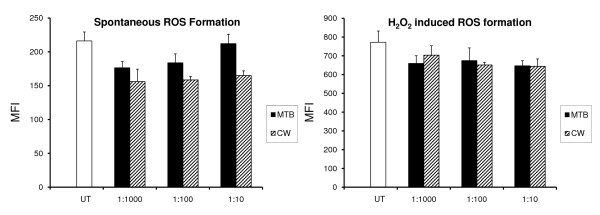
**The inhibition of formation of Reactive Oxygen Species (ROS) was evaluated in human polymorphonuclear (PMN) cells**. The DCF-DA mean fluorescence intensity in the cells was proportional to the oxidative damage. Untreated cells served as a baseline for oxidative activity in the absence of oxidative stress. The presence of BC fractions MTB and CW led to inhibition of the spontaneous ROS production in the cultures (**A**). H_2_O_2 _was used to induce oxidative stress in the cultures. Cultures treated with H_2_O_2 _in the absence of GBC30 fractions served as a positive control for ROS formation. Cultures treated with GBC30 fractions MTB and CW in the presence of oxidative stress showed reduced ROS formation compared to cultures treated with H_2_O_2 _alone (**B**). Data reflect averages of cultures performed in quadruplicate for each test condition. The data shown are representative of three independent experiments performed on PMN cells from different donors.

Both MTB and CW showed a clear inhibition of the spontaneous formation of reactive oxygen species in PMN cells (Figure 1A). The effect of CW showed a consistent inhibition of ROS formation across all doses tested, whereas the effect of MTB showed stronger anti-inflammatory effect at the lowest doses tested. The presence of MTB (1:1000) reduced spontaneous ROS formation by 20% (*p *< 0.004). CW (1:1000) showed a similar effect on lowering ROS formation (*p *< 0.005). At the 1:10 dilution of CW this effect was even stronger, resulting in a nearly 30% reduction in spontaneous ROS formation (*p *< 0.0008).

Treatment of PMN cells with MTB and CW before exposing the cells to oxidative stress and resulting ROS formation showed that both MTB and CW inhibited the H_2_O_2_-induced ROS formation (Figure 1B). MTB (1:100) and CW (1:100) reduced ROS formation by 15% and this reduction was statistically significant for CW (*p *< 0.03).

### Effect on PMN phagocytic activity

**Figure 2:**
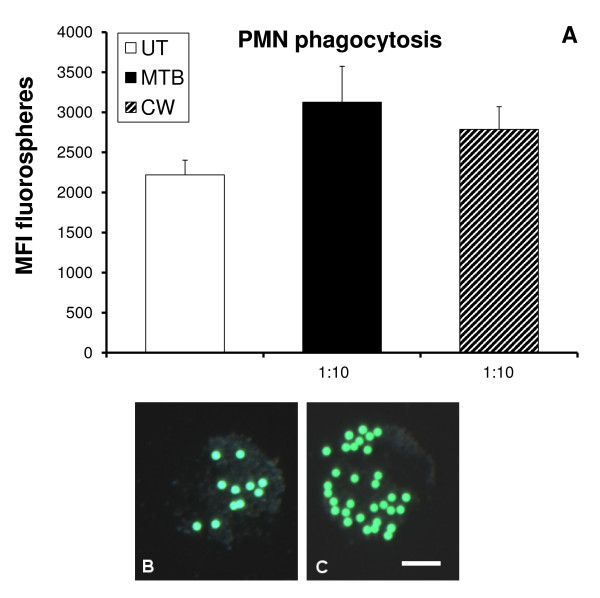
**Human polymorphonuclear (PMN) cells were evaluated for phagocytic activity, measured by the uptake of green-fluorescent beads**. (**A**) Flow cytometry analysis showed an increase in mean fluorescence intensity (MFI) of PMN cells treated with either MTB or CW. Microscopy showed that the level of bead uptake by an untreated PMN cell (**B**) was increased in PMN cells treated with CW (**C**). Photographs were taken at 600× magnification and the bar drawn in figure 2C represents 5 uM. The data shown are representative of PMN cells assayed in triplicate for each test condition.

Phagocytosis of microbial particles is an important part of the innate immune response. It is a rapid process, and the effect of a test product on enhancing this cellular function can be almost immediate. Phagocytosis was measured by how well PMN cells engulfed carboxylated fluorospheres. The mean fluorescence intensity (MFI) of phagocytic cells was then evaluated by flow cytometry. Exposure of PMN cells to MTB at the 1:10 dilution increased phagocytosis by 40% (Figure 2A). Exposure of PMN cells to CW at the 1:10 dilution increased phagocytosis by 25% (*p *< 0.02). Figure 2A shows the overall increase for the entire population of PMN cells, where some cells are phagocytic and others remain non-phagocytic. Figures 2B and 2C show the comparison of an untreated PMN cell to a PMN cell exhibiting increased phagocytosis following exposure to CW. Further dilutions of both products resulted in reduced PMN phagocytosis (*p *< 0.05) (data not shown).

### Differential effect on PMN cell random migration and chemotactic migration towards three chemo-attractants: f-MLP, IL-8, and Leukotriene B4 (LTB-4)

The PMN cell is a highly active and migratory cell type that plays a major role in immune surveillance. The migratory behavior of this cell type is divided into at least two types: a) random migration and b) directed migration. Random migration is part of normal immune surveillance, whereas directed migration happens towards specific chemoattractants.

The effects of MTB and CW on both types of migration were tested in parallel. Furthermore, the directed migration was tested towards three distinctly different chemotactic compounds: i) bacterial peptide f-Met-Leu-Phe (f-MLP); ii) the inflammatory cytokine Interleukin-8 (IL-8); and iii) Leukotriene B4 (LTB4).

**Figure 3:**
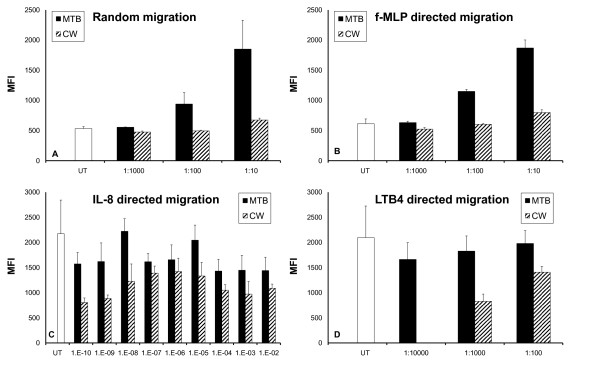
**Effects of GBC30 fractions on human PMN migration were evaluated using Transwell migration plates**. (**A**) Both MTB and CW treated PMN cells showed increased random migration behavior. (**B**) PMN cells treated with MTB or CW showed increased migratory behavior in response to the bacterial peptide f-MLP. (**C**) PMN chemotactic migration towards the cytokine IL-8 was decreased in the presence of MTB and CW. Extremely low doses of MTB and CW were tested for effects on IL-8 directed PMN cell migration. The cell wall fraction CW triggered inhibition of PMN cell migration towards IL-8 down to dilutions of 10^-10^. The metabolite fraction MTB showed similar but weaker inhibition across the same dose range. (**D**) PMN chemotactic migration towards the inflammatory mediator LTB4 was decreased by both the cell wall fraction MTB and the metabolite fraction CW, indicating an anti-inflammatory effect. The graphs show the averages and standard deviations of each culture condition performed in triplicate. The data shown are representative of three experiments performed on cells from different donors.

Both fractions of GBC30 induced the random migration of PMN cells. MTB (1:10) increased the random migration by 300% (*p *< 0.05), and CW (1:10) increased the random migration by 25% (*p *< 0.006) (Figure 3A). A distinct dose-dependent effect was seen with treatment of cells with MTB but not CW.

Both MTB and CW increased the migration towards the bacterial peptide f-MLP, implicating a support of anti-bacterial defense mechanisms (Figure 3B). Once again a dose-dependent response was seen following MTB treatment of cells. At the 1:10 dilution, MTB showed a 200% enhancement of f-MLP directed migration that was highly statistically significant (*p *< 0.0005). A 25% increase following CW treatment (1:10) was also statistically significant (*p *< 0.04).

Both MTB and CW reduced the IL-8 directed migration. Because we saw a strong reduction in IL-8 directed migration of PMN cells treated with low doses of CW and also saw some reduction with low doses of MTB, a dose study of IL-8 directed PMN migration with much lower doses of both MTB and CW was performed. As shown in Figure 3C, a reduction in IL-8 directed PMN migration was demonstrated at all dilutions of CW. This effect of CW was strongest at the 10^10 ^dilution, where migration was inhibited by over 60% (*p *< 0.03). MTB treatment of PMN cells at low doses also reduced IL-8 directed migration but not as much as CW. An interesting pattern of IL-8 directed PMN migration inhibition was seen with both MTB and CW. Neither product demonstrated a linear dose curve but rather intermediate doses (10^4 ^to 10^8^) of both MTB and CW showed less inhibition of IL-8 directed migration compared to higher or lower doses.

Both MTB and CW demonstrated a dose-dependent reduction in PMN migration towards LTB4 (Figure 3D). The 1:1000 dilution of CW inhibited migration by 60%. Cells treated with 1:1000 and 1:10000 dilutions of MTB also showed anti-inflammatory effects but these were not statistically significant.

### Induction of the CD69 activation marker on Natural Killer cells

Natural Killer (NK) cells are involved in our primary defense mechanisms against transformed cells and viruses. These cells travel in our blood stream in a state of rest, but can be immediately activated to a) kill cancer cells by either cell contact or secretion of cytotoxic compounds such as perforin and granzyme, b) proliferate, and c) secrete substances that attract other cells into the site. In order to investigate a possible effect of MTB and CW on NK cell activation, we examined changes in expression of the NK activation cell surface marker CD69. The increased expression of this marker has been associated with an increased cytotoxic activity of NK cells [30].

**Figure 4:**
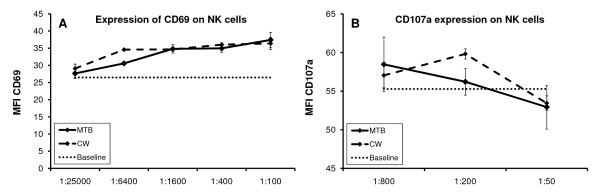
**Effects of the metabolite fraction MTB and cell wall fraction CW on NK cell activation status and cytotoxic activity was evaluated using human PBMC**. (**A**) Both MTB and CW fractions induced the expression of the activation marker CD69 on CD3 negative, CD56 positive NK cells in a dose-dependent manner. The effect was statistically significant for the 1:400 dilution of both MTB and CW. (**B**) Expression of CD107a on NK cells pretreated with MTB or CW showed a mild increase over baseline. The graphs show the averages and standard deviations of each culture condition performed in triplicate. The data shown are representative of three experiments performed on cells from different donors.

Both MTB and CW showed a clear dose-dependent induction of the expression of CD69 on NK cells (Figure 4A). This increase was statistically significant for the 1:400, 1:1600 and 1:6400 dilutions of both MTB and CW (*p *< 0.05). At the 1:400 dilution, CD69 expression was increased by 32% (*p *< 0.05) for MTB, and by 36% for CW (*p *< 0.006).

A mild increase in CD25 expression was seen on NK cells treated with a 1:100 dilution of MTB. No changes in CD25 expression on T cells were observed when comparing untreated cells to those treated for 18 hours with either MTB or CW (data not shown).

### Externalization of CD107a on NK cells in the presence of K562 tumor cells

One of the functions of NK cells is to kill tumor cells and virus-infected cells via cell-cell contact and by secretion of substances such as perforin. During this process, the CD107a receptor expressed on the interior of granules in the cytoplasm of NK cells is transiently brought to the cell surface. Thus, the transient CD107a expression on NK cells is a measure of their cytotoxic activity by secretion of cytotoxic substances. Figure 4B shows the change in mean fluorescence intensity (MFI) of CD107a expression on natural killer cells that have been exposed to tumor cells, with or without the addition of MTB or CW. Both MTB and CW show a mild increase in CD107a cell surface expression, with CW having the strongest effect at the 1:200 dilution, however the effect did not reach statistical significance.

### Effects on lymphocyte proliferation

With the question of immune support always comes the need to prove that a product would not on its own trigger exaggerated immune reactions. As part of a standard safety testing of natural products, we tested whether the test products had mitogenic potential, that is, whether they induce cell division in healthy human lymphocytes. Simultaneous to the test of mitogenic potential, we tested whether the test products had an effect on cells responsible for the adaptive immune defense, that is, T and B lymphocytes.

The GBC30 fractions were tested in serial dilutions in the absence and presence of mitogens. Two mitogens were tested in parallel: Phytohemagglutinin (PHA), which is a T cell mitogen that induces T cell proliferation, and Pokeweed Mitogen (PWM), which requires the collaboration of T cells, B cells and monocytes in the culture. Supernatants from the same cultures were processed for cytokine levels (see below).

Neither MTB nor CW had a mitogenic effect on lymphocyte proliferation following five days incubation at 37°C with product and culture media (data not shown). Both MTB and CW showed a reduction in lymphocyte proliferation in the presence of PHA and PWM. This reduction was statistically significant for both MTB and CW (*p *< 0.02) (data not shown).

### Effects on cytokine production

**Figure 5:**
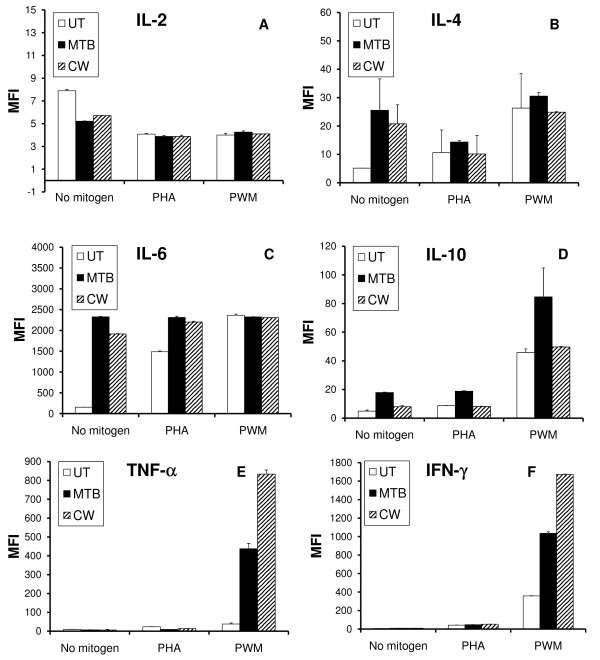
**The effects of the metabolite fraction (MTB) and cell wall fraction (CW) on cytokine production were evaluated on human PBMC cultures**. (**A**) Treatment of PBMC with MTB and CW resulted in reduced IL-2 production. There was no effect on mitogen-induced IL-2 production. (**B**) Treatment of PBMC with MTB and CW resulted in increased IL-4 production. There was no effect on mitogen-induced IL-4 production. (**C**) Treatment of PBMC with MTB and CW resulted in a strong increase in IL-6 production. Both MTB and CW further enhanced the PHA-induced IL-6 production. In contrast, neither MTB nor CW modulated the PWM-induced IL-6 production. (**D**) Treatment of PBMC with MTB, and to a lesser extent CW, resulted in an increased IL-10 production. MTB but not CW further enhanced the PHA-induced as well as the PWM-induced IL-10 production. (**E**) Both MTB and CW treatment of PBMC resulted in a mild reduction in TNF-α production, both in the absence of mitogens, and in the presence of PHA. In contrast, co-stimulation of PBMC with PWM and either MTB or CW resulted in a strong increase in TNF-α production. (**F**) Very slight increases in IFN-γ cytokine levels were seen following MTB or CW treatment in the absence of mitogens or presence of PHA. In contrast, co-stimulation of PBMC with PWM and either MTB or CW resulted in a strong increase in IFN-γ production. Cytokine production in PBMC cultures was assayed in duplicate. The concentration of MTB and CW in the assayed cell cultures was 1:100.

A flow cytometry-based Th1/Th2 cytokine bead array for the 6 cytokines IL-2, IL-4, IL-6, IL-10, TNF-α and INF-γ was used to evaluate the levels of cytokines present in the supernatants from 5-day lymphocyte proliferation cultures. Phytohemagglutinin (PHA) was used to induce T cell proliferation, and Pokeweed Mitogen (PWM) was used to induce T and B lymphocyte proliferation in a process that requires the collaboration of T cells, B cells and monocytes in the culture. Comparisons were made between untreated PBMC versus PBMC cultured in the presence of 1:100 dilutions of either MTB or CW, with and without PHA and PWM (Figure 5A-F). Untreated PBMC cells were cultured under identical conditions as MTB and CW treated PBMC cultures and cytokine levels in the untreated cultures used as a baseline for comparison to MTB and CW treated cultures. The relative changes of cytokines are shown, where "0" on the y axis indicates that treatment did not alter cytokine production from baseline.

In the absence of mitogens, both MTB and CW treatment of PBMC led to decreased IL-2 levels compared to untreated PBMC (Figure 5A). This reduction was statistically significant for MTB and CW (*p *< 0.02). No statistically significant changes in IL-2 levels were observed with MTB or CW treatment in the presence of either mitogen, compared to mitogen treatment alone.

In the absence of mitogens, both MTB and CW treatment of PBMC led to increased IL-4 levels compared to untreated PBMC (Figure 5B). This increase was statistically significant for MTB (*p *< 0.03). No statistically significant changes in IL-4 levels were observed with MTB or CW treatment in the presence of either mitogen, compared to mitogen treatment alone.

Both MTB and CW treatment of PBMC, in the absence of mitogens, led to massive induction of IL-6 production (Figure 5C). The increase was highly statistically significant (*p *< 0.003). No statistically significant changes in IL-6 levels were observed with MTB or CW treatment in the presence of Pokeweed mitogen, compared to Pokeweed mitogen treatment alone. The IL-6 induction by both MTB (*p *< 0.003) and CW (*p *< 0.002) in the presence of PHA was found to be highly statistically significant.

Both MTB and CW treatment of PBMC, in the absence of mitogens, led to induction of IL-10 production (Figure 5D). The increase was statistically significant (*p *< 0.02). PBMC treated with both MTB and PHA led to higher IL-10 production (*p *< 0.008) than if cells were treated with PHA alone. Treatment of PBMC with MTB and PWM also led to an increase in IL-10 production, however the data was not found to be statistically significant. No statistically significant changes in IL-10 levels were observed with CW treatment in the presence of either mitogen when compared to mitogen treatment alone.

In the absence of mitogens, TNF-α production was slightly lower than untreated PBMC in the presence of both MTB and CW (Figure 5E). This mild reduction was not statistically significant for either MTB or CW. Treatment of PBMC with either MTB or CW in the presence of PHA resulted in 2-fold decreases in TNF-α expression that were statistically significant for both MTB (*p *< 0.03) and CW (*p *< 0.006). In contrast, treatment of PBMC with MTB and CW in the presence of PWM resulted in strong increases in TNF-α levels. In the presence of PWM, MTB treatment produced an 11-fold increase (*p *< 0.03) and CW treatment a 22-fold increase (*p *< 0.02).

In the absence of mitogens, INF-γ levels increased in response to treatment with both MTB and CW (Figure 5F). MTB produced a 41% increase (*p *< 0.02) and CW resulted in a 54% increase (*p *< 0.02). Treatment of PBMC with either MTB or CW in the presence of PHA did not produce statistically significant changes in INF-γ expression. In contrast, treatment of PBMC with MTB and CW in the presence of PWM resulted in 3-fold (MTB) and 4-fold (CW) increases in INF-γ levels, both of which were statistically significant (*p *< 0.01).

**Figure 6:**
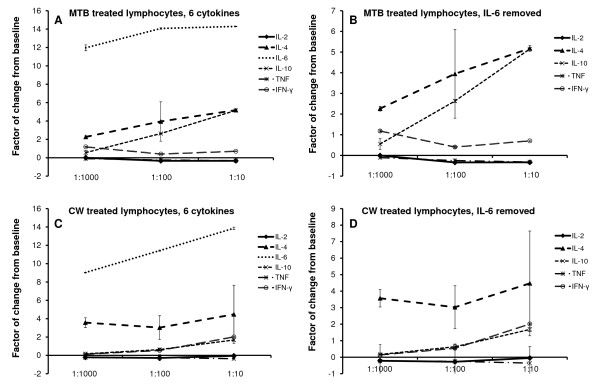
**The direct and dose-dependent effect of GBC30 fractions on Th1/Th2 cytokine production, in the absence of other stimuli, is shown**. The increase in production of the Th2 cytokines IL-4, IL-6, and IL-10, and composite effect on Th1 cytokines IL-2, IFN-γ, and TNF-α, is shown for the metabolite-rich fraction MTB (**A, B**) and the cell wall fraction CW (**C, D**). In the graphs **B **and **D**, the data from IL-6 were removed to better show the changes in production of the other 5 cytokines.

Thus, when evaluating the overall effect on production of all six cytokines, MTB induced an increased production of the three Th2 cytokines IL-4, IL-6, and IL-10 (Figure 6A). Given the strong increase in IL-6 production when cells were treated with MTB, a second graph shows the data on the five cytokines where data on IL-6 were removed (Figure 6B). Simultaneously, a reduced production of the two Th1 cytokines IL-2 and TNF-α was seen. The increase in all three Th2 cytokines was also seen for the CW fraction. However, Th1 cytokines were not reduced, and IFN-γ showed an increase above untreated cells (Figure 6C-D).

## Discussion

Inflammation, particularly chronic, low-grade inflammation, is an underlying phenomenon in many diseases, including cardiovascular disease, obesity, arthritic conditions, malignant transformation and progression of tumor growth, Alzheimer's disease, age-related cognitive decline, and depression. This has brought nutritional and nutraceutical anti-inflammatory products into focus, as people look for non-pharmacological ways to stay healthier longer [3].

Many types of bacterial cell wall components are known to interact with Toll-Like Receptors (TLR) 2 and 4 and thereby trigger pro-inflammatory immune defense reactions [31]. It was therefore particularly surprising that the GBC30 cell wall fraction, CW, showed inhibitory activity in several bioassays involving pro-inflammatory types of immune reactions. These included inhibition of ROS formation and reduced PMN cell chemotactic migration in response to IL-8 and LTB4.

The GBC30 culture supernatant, MTB, showed similar anti-inflammatory properties, although not quite as potent as CW. There were sufficient data sets showing distinctly different effects of MTB compared to CW. These included a more robust effect on increasing phagocytosis, enhancement of random and f-MLP directed PMN migration and induction of the cytokine IL-10 in 5 day PBMC cultures. Conversely, NK cell activation, demonstrated as an increase in CD69 expression, was nearly identical following MTB or CW treatment, across a broad dilution range. There were also multiple instances where MTB demonstrated a dose-dependent response that was not seen with CW, suggesting that with MTB treatment unique effects of the metabolites and not residual activity from contaminating CW was seen. Therefore, we conclude that even though some trace amount of cell wall components may be present in MTB, they do not account for the overall biological activity of MTB.

The effects of both GBC30 fractions were highly complex, and were not limited to a simple anti-inflammatory action. Both GBC30 fractions enhanced PMN cell random migration, which is an important aspect of immune surveillance. Both fractions also enhanced PMN cell migration in response to the bacterial peptide f-MLP, when used at higher doses. MTB treatment of cells had a greater effect on enhancing random and f-MLP directed PMN migration than CW treatment and this effect was dose dependent.

Both fractions induced the CD69 activation marker on human CD3-negative, CD56-positive NK cells, and also enhanced the transient expression of CD107a as an indication of increased NK cell cytotoxicity in the presence of transformed cells. The expression of CD107a as a consequence of degranulation of NK cells in the presence of NK sensitive tumor cells is transient, as CD107a is rapidly recycled by re-uptake into cytoplasmic granules containing cytotoxic substances. Thus, even though the CD107a data did not reach statistical significance, it supports the evidence of increased cytotoxic activity as indicated by increased expression of CD69. CD69 expression on NK cells is the only assay where MTB and CW performed nearly identical.

Such observations are normally associated with a more general activation of innate immune defense mechanisms, such as production of ROS, which was clearly not the case for the GBC30 fractions. Both MTB and CW inhibited spontaneous and H_2_O_2_-induced ROS formation. In the case of MTB, inhibition of spontaeous ROS formation was strongest at the lowest dilution tested. This interesting concentration-dependent effect of responses was also seen for both MTB and CW with regard to the migratory behavior of PMN cells in response to 3 different chemotactic stimuli. This suggests a complex immune response dependent on the concentration of the different BC30 fractions assayed. At high concentrations, a pro-inflammatory response is seen while at low concentrations an anti-inflammatory response predominates.

The effects on cytokine profile were tested in several ways, where both the direct effect, as well as the synergistic effects with other known lymphocyte activating compounds was studied. The products directly affected the cytokine levels *in vitro *by increasing the production of the Th2 cytokines IL-4 and IL-10. The shift towards Th2 cytokine production reflects support of adaptive immunity and antibody production, including sIgA secretion, which may enhance immune defense mechanisms along the gut mucosa [32]. The products may also affect important regulatory mechanisms affecting how the immune system reacts to food-borne microbes and antigens, such as induction of oral tolerance [33]. The products may have direct immune modulating effects on immune cells in lamina propria and Peyer's patches, affecting local and systemic cytokine levels and thus potentially affecting inflammatory conditions at locations unrelated to the gut environment.

A greater increase in IL-10 production occurred in cultures treated with MTB compared to cultures treated with CW. In the presence of the mitogens PHA or PWM, only MTB treatment produced an increase in IL-10 above baseline. IL-10 has an anti-inflammatory role in the gut and the consumption of IL-10 producing transgenic bacteria has shown efficacy in the treatment of Crohn's disease [34]. This suggests that consumption of GBC30 may also have a beneficial effect on conditions of chronic intestinal inflammation such as Crohn's disease and ulcerative colitis.

Both GBC30 fractions strongly induced production of IL-6 in PBMC cultures. IL-6 is not a simple pro- or anti-inflammatory cytokine, but helps to regulate inflammation. This regulatory cytokine has pleiotropic functions that are tissue-specific and dependent upon the physiological context [3]. The effects of IL-6 are influenced by whether it is present acutely or chronically. Thus, further evaluation of IL-6 levels in people consuming GBC30 is needed, in healthy individuals, individuals experiencing acute infection, in those with chronic inflammatory conditions, insulin resistance, and endothelial dysfunction. Based on the complexity of the *in vitro *effects of both GBC30 fractions, we suggest that consumption of the probiotic strain may show protective effects in both acute and chronic conditions.

A mild reduction of IL-2 and TNF-α was seen, along with an increase in IFN-γ production. The pro-inflammatory functions of TNF-α and IFN-γ are undisputed; however IL-2 has complex and opposing roles during the induction and termination of inflammatory responses. In contrast to the direct anti-inflammatory effect on cytokine profile, GBC30 strongly enhanced responses to the known stimulus Pokeweed mitogen (PWM), which requires the collaboration of macrophages and different lymphocyte subsets. The increase in IFN-γ in PWM-treated cultures following treatment with MTB and CW is of interest regarding the role of IFN-γ producing dendritic cells in mucosal immunity against orally acquired pathogens [35]. This suggests that GBC30 compounds may enhance the response to invading pathogens in, for example, Peyer's Patches, where different cell types including dendritic cells collaborate on producing innate and adaptive immune defense reactions.

## Conclusions

The complex actions of both GBC30 fractions include nti-inflammatory effects, while also supporting key aspects of innate immune defense mechanisms. If some or all of the effects observed *in vitro *can also potentially take place when GBC30 replicates in the intestinal environment, this could have important implications, not only for protection of the host from potentially pathogenic bacteria, but even more importantly, for controlling local inflammatory processes and protecting epithelial integrity, which then allows proper separation of gut lumen, and proper nutrient assimilation and antigen presentation to the immune system. The data suggest that further clinical work should be initiated to evaluate the effect of consumption of GBC30 as well as the MTB and CW fractions on the control of inflammatory reactions *in vivo*, while simultaneously supporting anti-bacterial, anti-viral, and anti-tumor defenses *in vivo*.

## Abbreviations

DCF-CA: Dichlorofluorescein Diacetate; DMSO: Dimethylsulfoxide; f-MLP: formyl-met-leu-phe; GBC30: GanedenBC^30™ ^*Bacillus coagulans*: GBI-30, PTA-6086; IL: Interleukin; LTB4: Leukotriene B4; MFI: Mean Fluorescence Intensity; NF-κB: Nuclear Factor κB; NK: Natural Killer Cells; NKT: Natural Killer T Cells; PBMC: Peripheral Blood Mononuclear Cells; PBS: Phosphate Buffered Saline; PHA: Phytohemagglutinin; PMN: Polymorphonuclear Cells; PWM: Poke Weed Mitogen; ROS: Reactive Oxygen Species; RPMI: Roswell Park Memorial Institute Culture Medium; TLR: Toll-like Receptors; TNF: Tumor Necrosis Factor.

## Competing interests

Ganeden Biotech, Inc. sponsored the study that is being reported. AIBMR Life Sciences and NIS Labs were paid for the conception, design and execution of the *in vitro *study, as well as for the preparation of the manuscript. Neither organization has any financial interest in Ganeden Biotech, Inc.

## Authors' contributions

GSJ and JRE conceived of the idea to test and compare the bioactivity of bacterial cell walls and metabolites. GSJ and KFB planned the procedure for generating the two test fractions. GSJ designed the study and supervised the lab work and data analysis. KFB performed the production of the two fractions. KFB and SGC performed the in vitro testing, analysis, and contributed to the writing of the manuscript. KFB did the statistical analysis. GSJ and JRE finalized the manuscript writing. All authors have read and have approved the final manuscript.
